# A novel form stable phase change material with comb-like cross-linked polyurethane as supporting skeleton

**DOI:** 10.1038/s41598-022-21640-3

**Published:** 2023-03-31

**Authors:** Yunyun Yang, Shenghua Xiong, Ju Fu, Yuanhua He, Yi Wu, Yi Xu

**Affiliations:** 1 College of Civil Aviation Safety Engineering, Civil Aviation Flight University of China, Guanghan, 618307 China; 2 Civil Aircraft Fire Science and Safety Engineering Key Laboratory of Sichuan Province, Civil Aviation Flight University of China, Guanghan, 618307 China

**Keywords:** Chemistry, Polymer chemistry, Materials science, Materials for energy and catalysis

## Abstract

To improve the homogeneity of phase-change materials (PCMs) composites for thermal energy storage, the poly(ethylene glycol monomethyl ether)-based trimethylolpropane (Ymer-N120) with long side ethyoxyl chains is employed to form comb-like polyurethane which functioned as supporting materials for PCMs. And the results of Fourier transform infrared spectroscopy (FTIR), X-ray diffraction, differential scanning calorimetry, accelerated thermal cycling testing, thermogravimetric analysis and field emission scanning electron microscopy (FESEM) suggested a crosslinked polyurethane embedded with micron grade myristic acid (MA) crystals was prepared during the thermal curing process. The obtained comb-like polyurethane (YP) can provide 3D structure supporting materials for melting MA. And the long side ethyoxyl chain of Ymer-N120 promote the melting MA form micron-sized crystals. The results of thermal reliability testing confirmed the advantages of same methylene groups in side chains and suggested the maximal hold capability of YP crosslinks is about 50 wt% of composites. With the 50 wt% addition of MA, YPM50 can supply high latent heat (over 90 J/g of YPM50) with fine thermal stability (due to its initial decomposing temperature reaches 190 °C) without leakage (after 500 times of accelerated thermal cycling testing). All results indicated this structure supplies an effective solution for the leakage of PCMs, which show a promising application in TES.

## Introduction

The exploration and utilization of energy have become the crucial and urgent issue as the earth are suffering the energy crisis and environment pollution^1–3^. Thermal energy storage (TES) technology can solve the time and spatial mismatch between energy demand and supply^4^, has be employed to harvest the renewable energy^5–8^ and collect the domestic/industrial waste energy^9,10^. Phase change materials (PCMs) can reversibly store and release the thermal energy by the melting and crystallization of crystalline materials^11,12^, have attracted extensive attention for TES^13^ systems in following fields: constructions and buildings^13–15^, solar energy storage^16–18^, geothermal energy storage^4^, battery thermal management system (BTMS)^12,19^ and other thermal management system systems. Due to the high energy density, negligible temperature and volume variation of PCMs during the energy storage process, PCMs such as fatty alcohol^20^, polyethylene glycol^21^, paraffin^22^ and fatty acid^23^ have been extensive used in many fields like smart buildings, aerospace, smart cloths and industrial heat waste recovery etc.

Among the PCMs, the fatty acids with long flexible chain have been thoroughly studied due to their adjustable phase-change temperature, nontoxic, and chemical stability^24,25^. However, the fatty acids suffer the leakage above the melting temperature (*T*_m_) ^26^, which may cause the TES system failure, equipment pollution even fire hazard, and has restricted the development of fatty acids. Therefore, the fatty acids are always encapsuled by porous supporting materials^27^ like carbon nanotube (CNT), graphene, expanded graphite etc. to fabricate as form stable PCMs (FSPCMs) to prevent the leakage of fatty acids even above *T*_m_. For example, Hu^28^ has reported a fatty acids eutectics based FSPCMs, showing excellent energy storage ability and superior shape-stabilization, by employing reduced graphene oxide/carbon nano-felts supporting materials.

However, the FSPCMs suffer poor stability and reliability due to the inferior interface interaction of fatty acids and supporting materials, which may cause FSPCMs failure once the supporting skeleton was suffer external force and chemical solvent. Employing polymer matrix as the supporting materials is one of the most efficient ways to improve the stability and reliability of fatty acids based FSPCMs. The polymer networks especially the crosslinking polymer networks can effectively constraint the leakage of melted fatty acid when the temperature is higher than the melting temperature of fatty acid^29^. For example, Pandey^30^ prepared a porous amphiphilic polymeric matrix with high phase transfer repeatability and long durability due to its good water dispersibility of polymeric particles. However, the interface incompatibility between the fatty and polymer matrix will occur after multiple repeatedly phase change process, and resulting in the effusion of fatty acids. Therefore, it is necessary and interesting to fabricate a stable and reliable FSPCMs by enhancing the interaction between fatty and polymer matrix^25,31^. The comb-like polymer with side alkyl chains have been studied because of their long side chains’ interaction with PCMs. Yao^32^ used a comb-like structural phase-change supporting material (PPEGMA) to supply tightly intertwines among the side long chains of polymer and PCMs chains by the action of induced dipole force.

In this work, a crosslinking polyurethane (named YP) was prepared via brief thermal curing of polyaryl polymethylene isocyanate (PAPI) as coupling agent and poly(ethylene glycol monomethyl ether)-based trimethylolpropane (Ymer-N120) with flexible chain as polyol. Meanwhile, the long flexible chain of Ymer-N120 became the side chains of YP. The fabricated comb-like YP networks functioned as supporting skeleton to capsule myristic acid (MA) for thermal energy storage. And the long side chains favor to the enhancement of the interaction between the MA and polymer matrix, which improve the distribution of MA. This strategy shows distinct advances including compact synthetic route, clean preparation process without organic solvents or generation of toxic gases. In addition, the chemical structure, crystalline performances, phase-change performances, thermal reliability, thermal stability and microscopic morphology of cured FSPCMs were extensively analysed. All the results suggested this comb-like structure with long flexible chains afford a potential solution for the leakage of melting MA.

## Sample preparation and characterization

### Materials

Poly(ethylene glycol monomethyl ether)-based trimethylolpropane (Ymer N120, Mw = 1000 g/mol) was purchased from Perstorp. Myristic acid (MA, CAS: 544-63-8) was afforded by Kelong Chemical Reagent (Chengdu, China); Polyaryl polymethylene isocyanate (PAPI, Mw = 381 g/mol; NCO wt% = 33.07; average functionality is 3) was purchased from Yantai Wanhua polyurethane Co., Ltd. (Shandong, China). All the materials were used without being treated.

### Preparation of polyurethane composites

**Figure 1:**
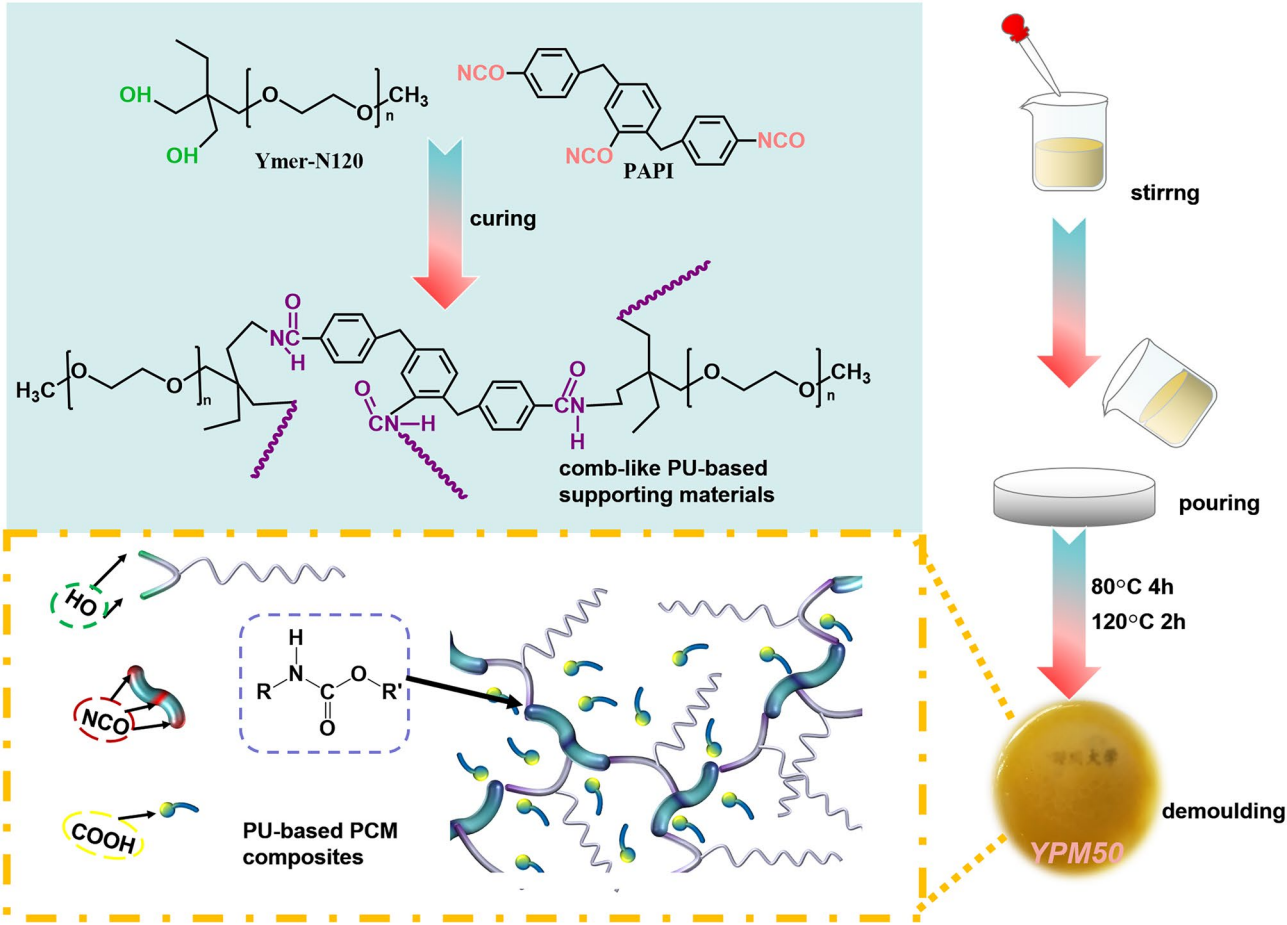
The curing route of YPM50 phase-change composites.

3.00 (± 0.01)g (0.003 mol) dried Ymer N120, 0.76 (± 0.01)g (0.002 mol) PAPI and different amount MA were decanted into the flask, and blended by the mechanical stirring about 10 min to form a homogeneous mixture at 80 °C. After degassing, the mixture was transferred into a tetrafluoroethylene plate and placed in an 80 °C vacuum oven. The mixture was heated for 4 h at 80 °C, and next heated at 120 °C for 2 h, its curing route was shown in Fig. 1, and the crosslinked polyurethane (PU) materials was named as YP. According to the addition of MA, the obtained PU-based PCM composites were designated as YPM50 (YP added 50 wt%MA), and YPM60 (YP added 60 wt%MA), respectively.

## Results and discussions

### Chemical and crystalline structure of PCM composites in comb-like supporting skeleton

**Figure 2:**
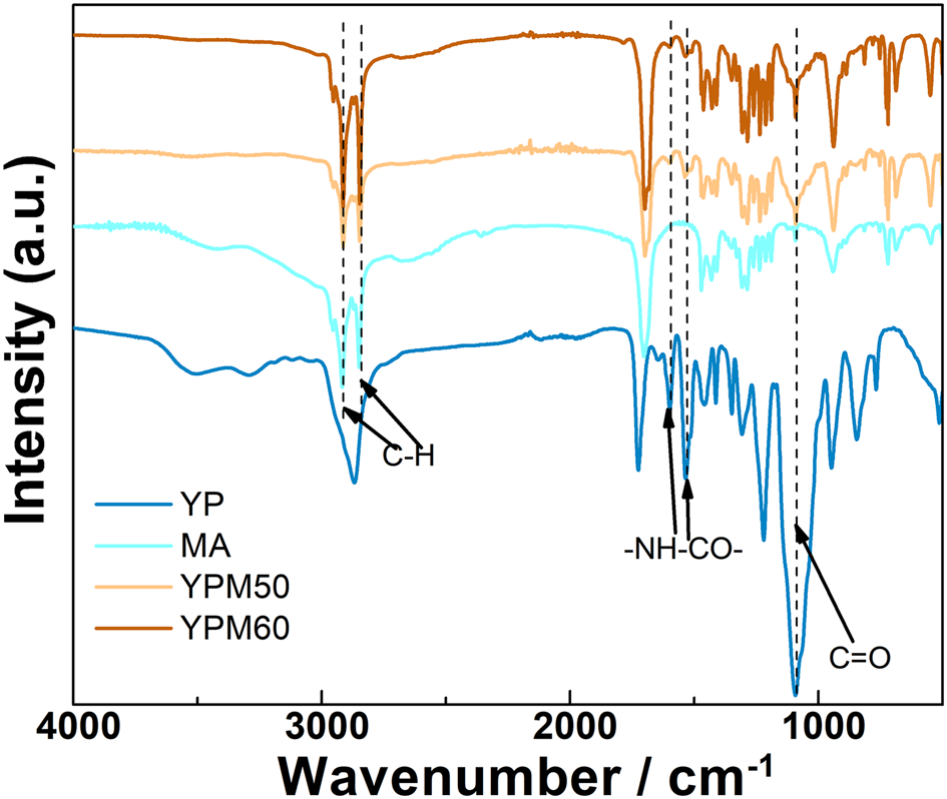
The FTIR spectra of YP, MA, YPM50 and YPM60.

The FTIR testing was firstly used to investigate the chemical structure and reaction of Ymer-N120 and PAPI, and the addition of MA in comb-like crosslinks. As shown in Fig. 2, the apparent absorption peaks of –NH–COO– around 1598 cm^−1^ and 1534 cm^−1^ in YP testify the polycondensation of Ymer-N120 and PAPI. The –NH–COO– absorption peaks also appear in YPM50 and YPM60 also indicate that the reaction of Ymer-N120 and PAPI is not interrupted by addition of MA. Besides, obvious peaks around, 1097 cm^−1^, 963 cm^−1^, 842 cm^−1^ and 766 cm^−1^ belonging to the C–O–C group, –CH_2_– and –CH_3_ groups are also appeared in YPM50 and YPM60. Furthermore, two characteristic peaks marked by dash lines at around 2913 cm^−1^ and 2847 cm^−1^ are belong to the methylene of MA are also observed in YPM50 and YPM60. The results imply that YP and MA based PCMs were successfully prepared by one-step polymerization.

**Figure 3:**
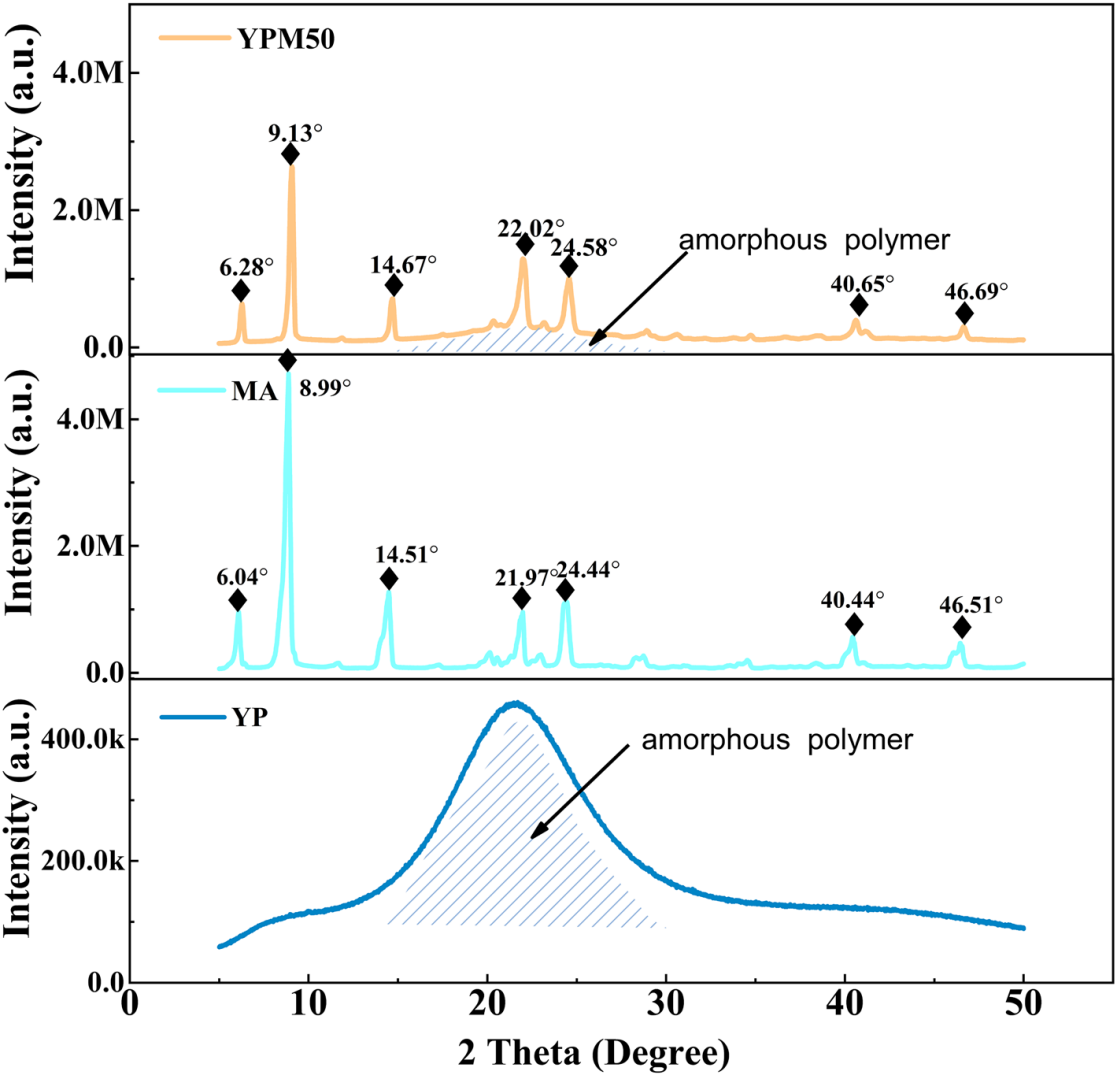
The XRD diffractogram of YP, MA and YPM50.

The crystalline structure of YP, MA and YPM50 are studied by XRD, as shown in Fig. 3, a blunt and broad peak at 21.6° is detected in YP indicating the amorphous structure of YP. MA crystal exhibits seven sharp peaks at 6.09°, 8.99°, 14.51°, 21.97°, 24.44°, 40.44° and 46.51°. The seven similar sharp peaks were also observed in YPM50, which indicated the excellent crystalline performance of MA even encapsuled by YP. The almost same peak position of pure MA and YPM50 suggest the long flexible chains of Ymer-N120 cause aliphatic MA molecules easy to form orderly crystalline microspheres when the temperature is below the freezing point of MA. The negligible differentiation of diffraction angle and *d*-spacing of the crystal planes implies the inherent crystalline properties of MA have not been affected by the polymer skeleton. Compared with pure MA, YPM50 shows lower peak intensity due to the addition of polymeric skeletons in PCM composites. The method for estimating the partial solubility parameters of polymers and pure organic compounds is established from group contributions. The equation for the estimation of δ is as follows:

$$ \updelta  = \frac{{\sum {F_{i} } }}{{{\tilde{\text{V}}}}} = \sum {F_{i} } *\frac{\rho }{{M_{W} }} $$

where F_i_ are the group contributions of type i to the molar gravitational constant, ρ and M_w_ is the density and molecular weight of polymers and pure organic compounds. According to the above equation, the solubility parameters of YP is 7.701, is very close to that of MA (7.558). The close solubility parameters of YP and MA suggested the good compatibility.

### Phase-change properties of YPM

**Figure 4:**
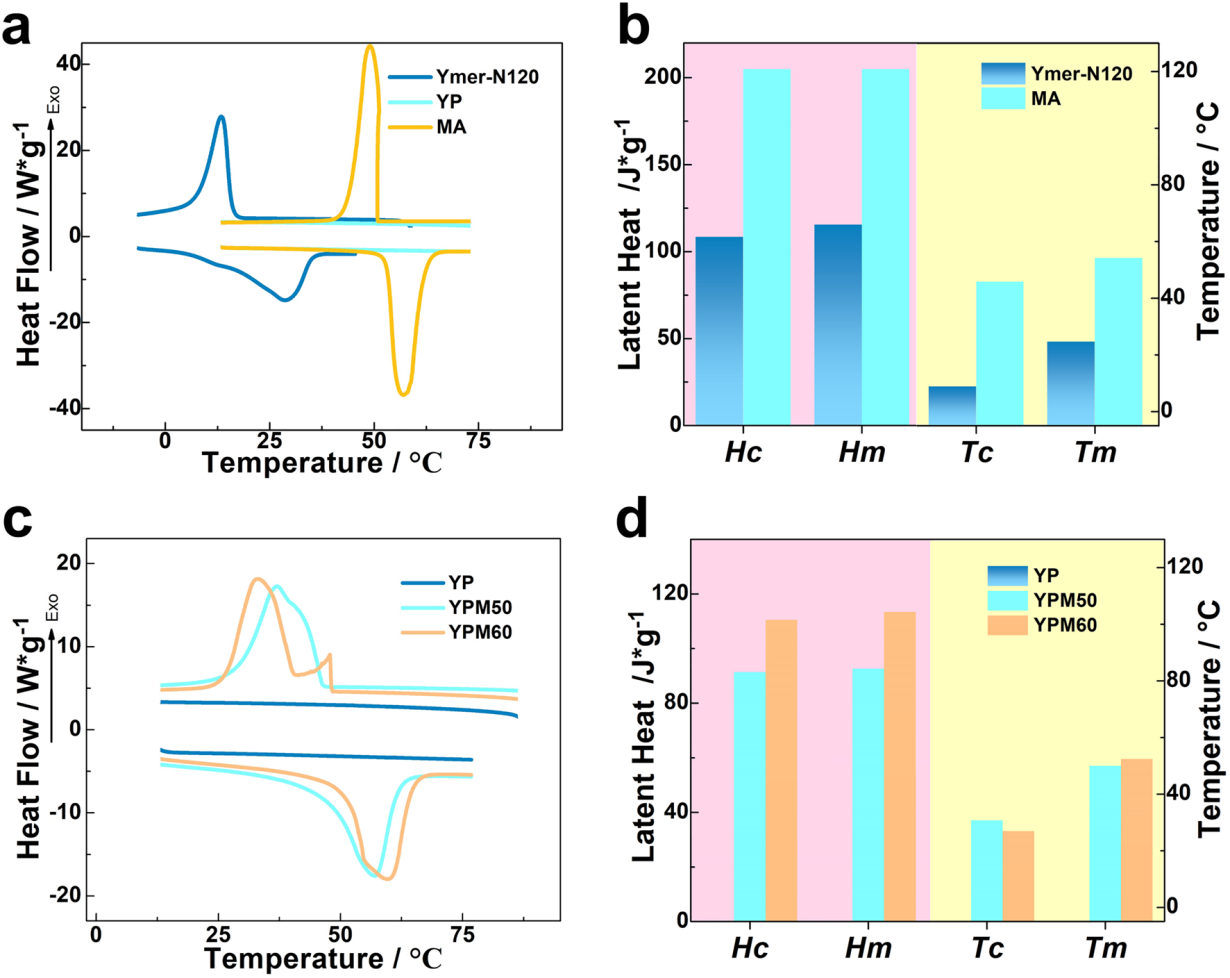
The (**a**) DSC curves of Ymer-N120, YP and MA (**b**) phase change properties of Ymer-N120 and MA (**c**) DSC curves of YP, YPM50 and YPM60 (**d**) phase change properties of YP, YPM50 and YPM60.

The phase-change properties, brought by the reversible crystallization and melting of crystalline segments, are the predominant properties of PCMs. The phase change temperature, including crystalline temperature (*Tc*), melting temperature (*Tm*), crystalline enthalpy (Δ*Hc*) and melting enthalpy (Δ*Hm*), were measured via DSC analysis in this part. As shown in Fig. 4a, Ymer-N120 exhibits obvious crystalline performance, and has high latent heat [115.6 J/g of Δ*Hm*, 108.5 J/g of Δ*Hc* (Fig. 4b)]. The incorporation of PAPI will restrict the crystalline properties of Ymer-N120 segments, reflecting by no peak is detected in the testing range for YP, which is consistent with the XRD results. Due to the excellent phase change properties (205.0 J/g of Δ*Hm* and 205.0 J/g of Δ*Hc*) of MA, the YPM50 and YPM60 have obvious endothermic peaks and exothermic peaks which are similar to those peaks of MA during the heating and cooling cycles (Fig. 4c). YPM50 has an energy storage ability 92.6 J/g of Δ*Hm* and 91.3 J/g of Δ*Hc,* YPM60 shows the higher latent high with 113.4 J/g of Δ*Hm* and 110.5 J/g of Δ*Hc* (Fig. 4d). Compared with pure MA, the decreasing functional phase change ingredient cause lower latent value in YPM50 and YPM60. Besides, the solution effect and hindered effect of polymeric skeletons also restrict crystalline capability of MA. The phase change temperature (*Tc* and *Tm*) determine the application temperature-range of PCMs, the phase-change temperature range of YPM50 and YPM60 is about 33.1–57 °C, which is suit for application in smarting building and cloths. Compared with the *Tc* (49.0 °C) and *Tm* (57.0 °C) of MA, the *Tc* of YPM50 and YPM60 are 37.1 °C and 33.1 °C, the *Tm* of YPM50 and YPM60 are 57.0 °C and 59.5 °C, correspondingly. The slightly change of *Tc* of YPM50 and YPM60 is caused by the solution effect of the flexible side chains. In summary, the application temperature range has also been broadened by the comb-like PU crosslinks.

**Figure 5:**
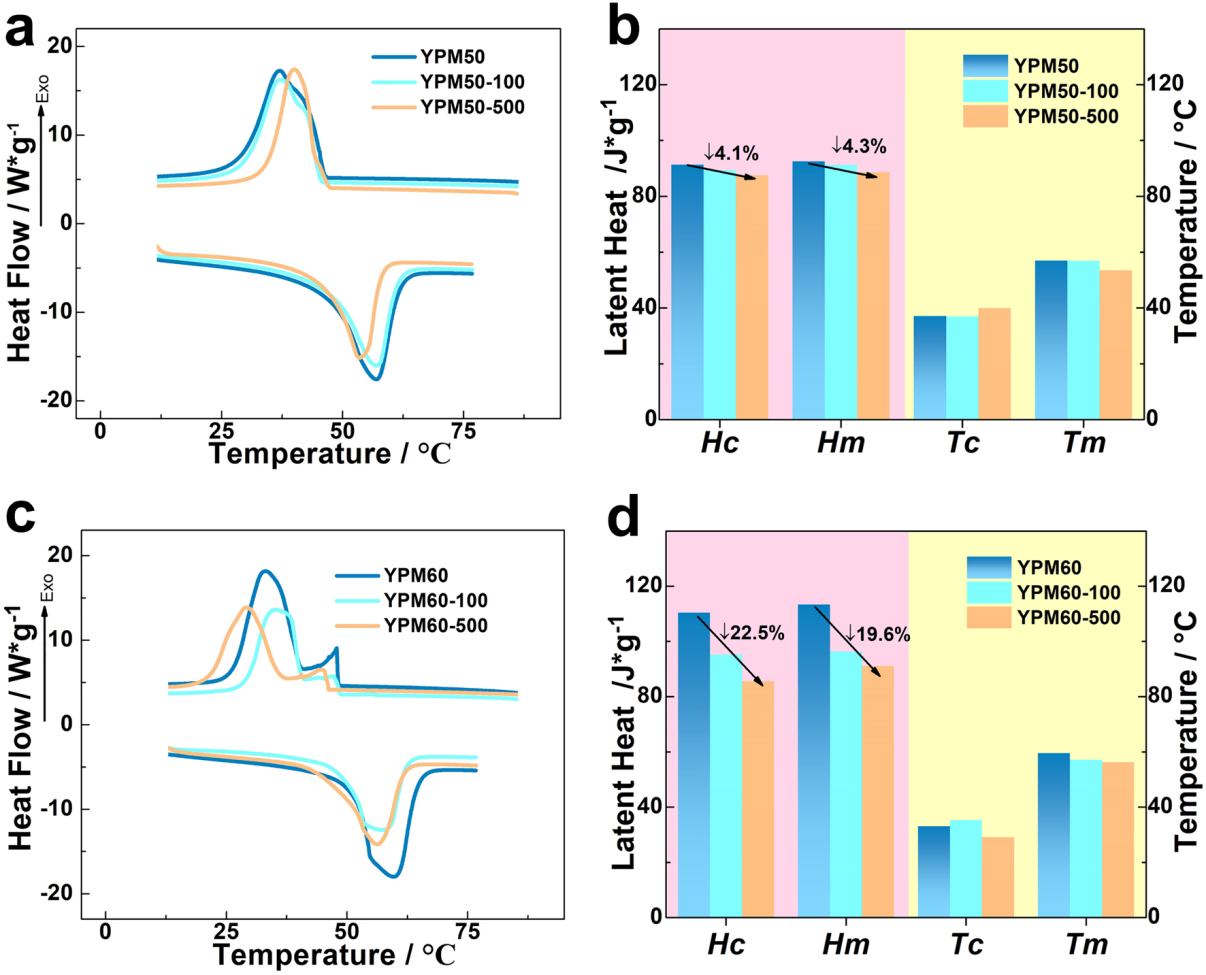
The (**a**) DSC curves of YPM50, YPM50-100 and YPM50-500 (**b**) phase-change properties of YPM50, YPM50-100 and YPM50-500 (**c**) DSC curves of YPM60, YPM60-100 and YPM60-500 (**d**) phase-change properties of YPM60, YPM60-100 and YPM60-500.

The thermal reliability is an effective factor, and will determine the service life of PCMs. The thermal reliability of YPM50 and YPM60 is also evaluated by accelerated thermal-cycles and DSC testing, the related results are showed in Fig. 5a,c, and the data of phase-change properties are synchronously listed in Fig. 5b,d. The thermal treated PCM composites exhibit the semblable single exothermic and endothermic peaks of original sample. The YPM50-500 (YPM50 was thermal treated by 500 cycles of heating–cooling processes) solidifies at 39.98℃ with 87.6 J/g freezing latent heat, and melts at 53.5 °C with 88.6 J/g melting latent heat, which are slightly shifted compared to those of YPM50. The small decrease of latent heat and phase-change temperature imply that the phase-change properties of YPM50 are not influenced by the 500 times of thermal-treating due to the good coating ability of YP crosslinking networks. While the *Tc*, *Tm*, Δ*Hc* and Δ*Hm* of YPM60-500 is about 29.16 °C, 56.26 °C, 85.6 J/g (with 22.5% decrease) and 91.1 J/g (with 19.6% decrease). Those big decreases are resulted from the leakage of melting MA. The better thermal reliability of YPM50 suggests the optimum addition of MA is 50 wt%.

**Figure 6:**
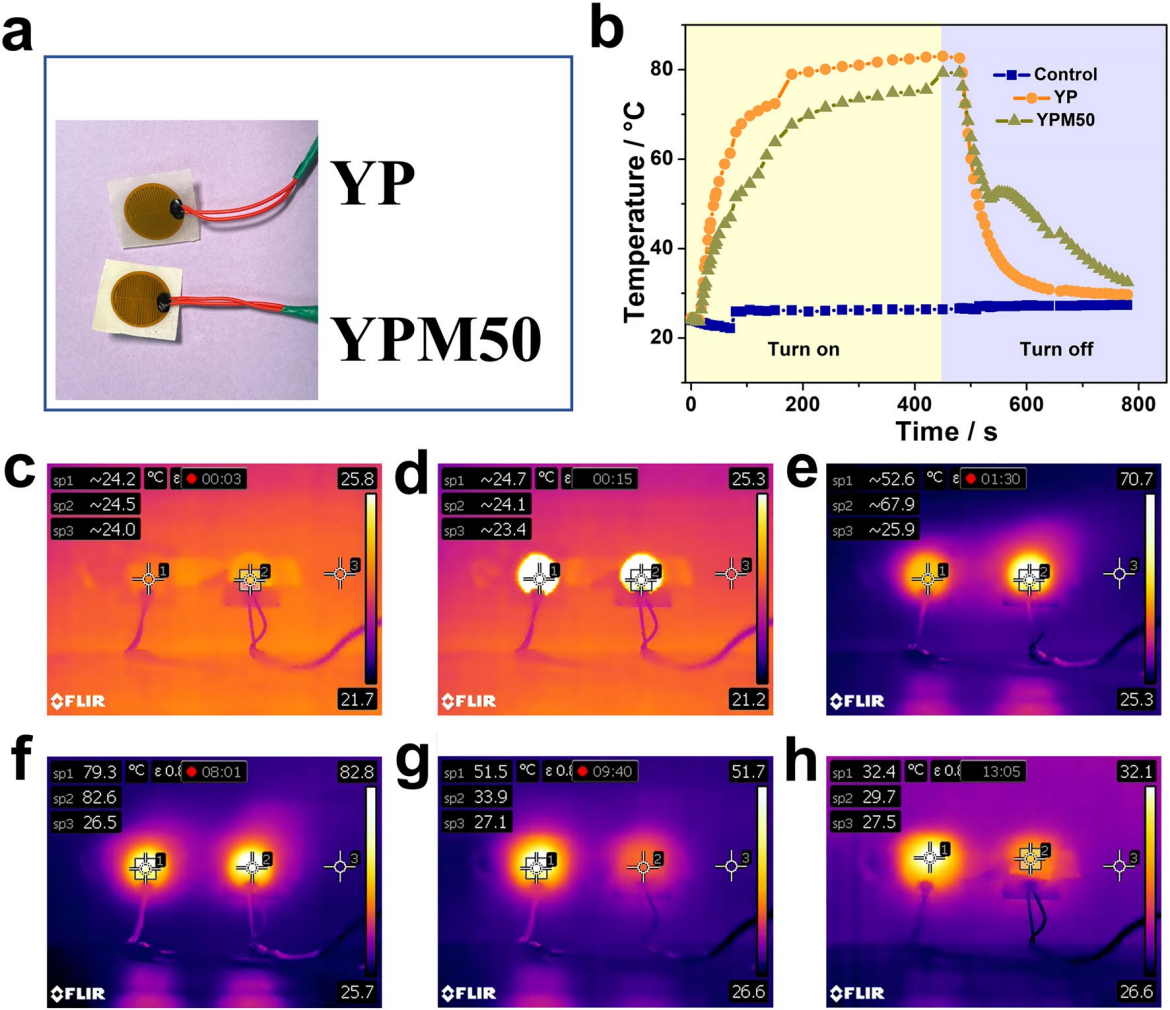
The (**a**) thermal management layer (**b**) temperature evolution curves of the samples during heating and cooling, Control sample was the ambient temperature; the infrared thermal imagines(sp1 is the temperature of PI-based heater with YPM50 film, sp2 is the temperature of PI-based heater with YP film, sp3 is the ambient temperature during testing) (**c**) under room temperature (**d**) at heaters turning on (**e**) at the rapid upward (**f**) at heaters turning off (**g**) at the rapid decline (**h**) after turning off 304 s.

Moreover, a simple device (see Fig. S1 in Supplementary Information) with a PI-based heater (50 mm, 2.7Ω) as heat source was employed to further evaluate the potential application of our samples in the thermal management field. Same size of YP and YPM50 films were used as the thermal management layer (Fig. 6a). Meanwhile, the infrared thermal camera (FLIR T420) was used to record the real-time temperature of the heater during use (see the real-time video in Supplementary Video 1). As depicted in Fig. 6b, at beginning the temperature of three point (sp1 is the temperature of PI-based heater with YPM50 film, sp2 is the temperature of PI-based heater with YP film, sp3 is the ambient temperature during testing) are almost same(Fig. 6c), when the heaters were turned on (Fig. 6d), the temperature of YP rapidly rose up to 67.9 °C within 75 s; however, after being integrated with MA, the heating rate of the YPM50 was obviously slowed, and the ultimate temperature is 52.6 °C after heating for 75 s (Fig. 6e). Then the heater was turned off (Fig. 6f), the temperature of YP decreased 59%, while that of YPM50 decreased 35% (Fig. 6g). And after 304 s, the temperature of PI-heater with YPM50 was still higher than that of PI-heater with YP (Fig. 6h). Those results indicated that our YPM50 possess promising prospects in the thermal management field.

### Thermal properties and microstructure of PU-based PCM composites

**Figure 7:**
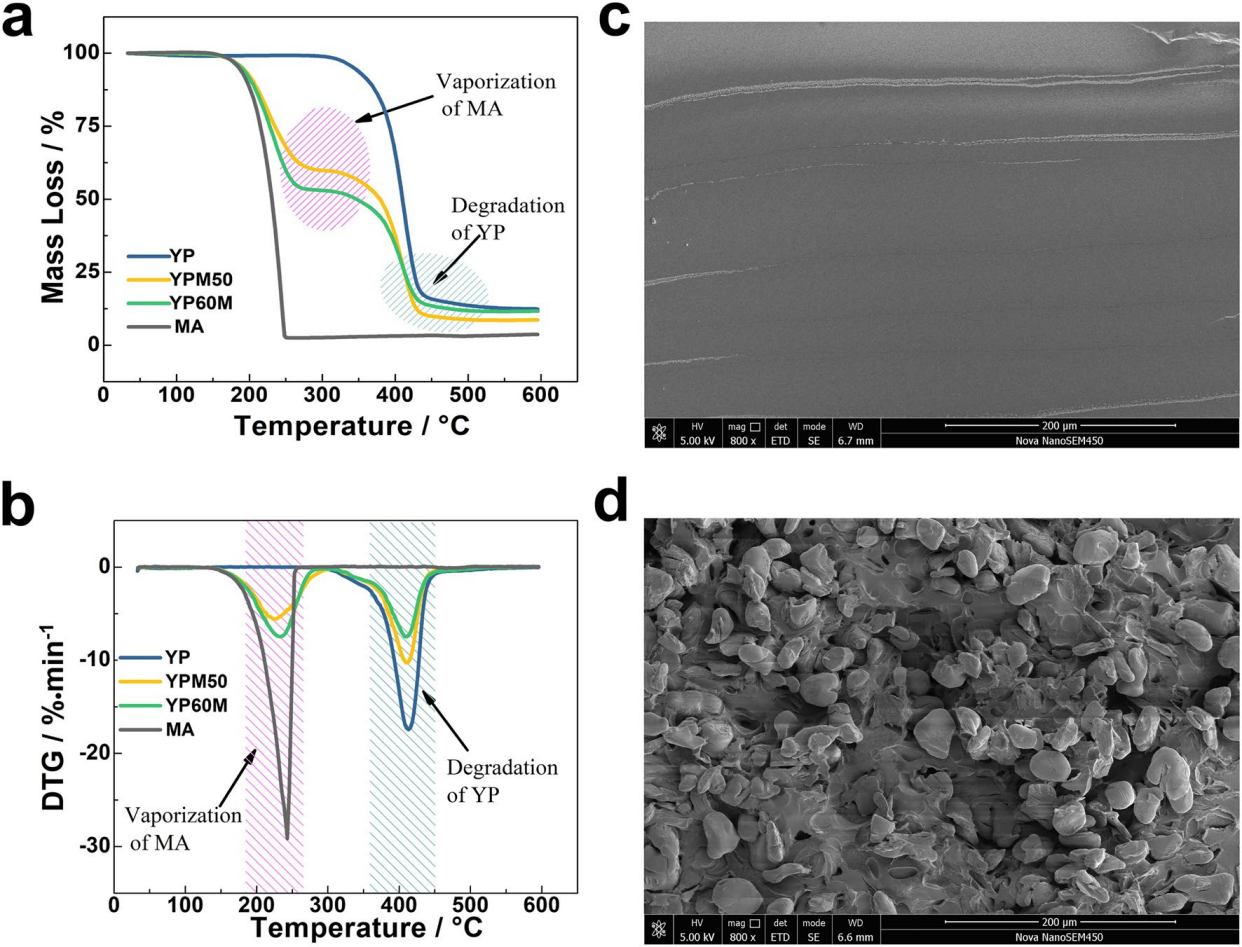
The (**a**) TGA curves of YP, YPM50, YPM60 and MA (**b**) DTG curves of YP, YPM50, YPM60 and MA (**c**) SEM images of YP (**d**) SEM images of YPM50.

The thermal stability of prepared PCM composites is also studied with TGA. The related TGA and DTG curves are listed in Fig. 7. In Fig. 7a, the MA occurs mass loss at 180℃, which is caused by the vaporization, while YP has distinguished thermal stability due to its decomposition temperature exceeds 310℃. With the addition of MA, YPM50 and YPM60 samples have a previous mass loss peak at the boiling temperature of MA, while YP shows one-step degradation mechanism (see Fig. 7b). As listed in Table 1, the initial mass loss temperature of YPM50 and YPM60 is slightly higher than that of pure MA, indicating the protection effect of YP networks on MA. Besides, the initial thermal-degradation temperature of YPM50 and YPM60 is quite higher than their phase-change temperature, suggesting that the thermal stability completely satisfy the required phase-change temperature. YP and YPM50 were cooled in liquid nitrogen for 20 min, then were broke in liquid nitrogen. The fracture surface was sprayed with gold to further study the dispersion of MA in YP networks by SEM. The surface morphology of YP and YPM50 are shown in Fig. 7c,d, respectively. The YP exhibits a smoothly surface, while the YPM50 shows a rough and pockmarked surface with obvious detection of granular MA crystal (about 30–50 μm) dispersion in YP matrix. The fine distribution of MA suggests the crosslinks with long flexible chains act as excellent supporting materials for PCM composites. The SEM figure of YPM50 after 500 thermal cycles of heat-cool cycle in a temperature-controlled chamber was attached in Fig. S2 (Supporting Information). There are morphological changes of YPM50-500. The micron particles of MA are getting fainter and smaller. Those changes suggest there is tightly intertwines between the side long chains of YP and MA by the action of induced dipole force. After times thermal cycles, the interface of YP and MA is wakening. And the decreasing of enthalpy also confirmed the dissolving capacity of side chains from YP. And this kind of structure supports a promising prevention and control strategy for the leakage MA above the melting temperature.

**Table 1:** The related TGA and DTG data of YP, YPM50, YPM60 and MA under nitrogen.

Samples	*T*_5%_ (°C)^a^	*T*_50%_ (°C)^b^	*T*_*PL*_ (°C)^c^	*P*_*L*_ (%/min)^d^	*T*_*PH*_ (°C)^e^	*P*_*H*_ (%/min)^f^
YP	343.3	410.1	–	–	413.8	− 17.45
MA	185.9	230.8	243.3	− 29.43	–	–
YPM50	190.5	379.0	225.2	− 5.71	411.3	− 10.27
YPM60	189.0	347.0	233.5	− 7.48	410.1	− 7.47

^a^The testing temperature when the mass-loss of sample reaches 5 wt%, defined as the initial thermal-degradation temperature.

^b^The testing temperature when the mass-loss of sample reaches 50 wt%.

^c^The testing temperature of mass-loss peak at low temperature (the range of 0–300 °C).

^d^The mass-loss rate of the mass-loss peak at low temperature (the range of 0–300 °C).

^e^The testing temperature of mass-loss peak at high temperature (the range of 300–600 °C).

^f^The mass-loss rate of the mass-loss peak at high temperature (the range of 300–600 °C).

## Conclusions

A novel FSPCM YPM50 was prepared for TES through using the crosslinking PU with flexible side chain as supporting skeleton and MA as phase-change functional segments. The crosslinked networks can prevent the leakage of MA above the melting temperature and the flexible side chain favor to improving the distribution of MA in fabricated YPM50. The FTIR results demonstrated that PU crosslinks were successfully synthesized via one-step polymerization, and the crosslinking networks were not affected by the addition of MA. Due to the solvent effect of flexible side groups in polyurethane networks, the MA crystal was well encapsulated and even distributed. And the latent heat of YPM50 and YPM60 exceed 90 J/g and 110 J/g, meaning an excellent energy storage ability which is much attraction for the TES. Furthermore, YPM50 also shows a good thermal reliability even the after 500 times thermal treating due to the protection of flexible side chains on MA. The SEM results suggested the granular MA is evenly dispersion in crosslinking networks. And this structure supports a promising prevention and control strategy for the leakage MA even above the melting temperature of MA.

## Methods

The chemical structure of YP, YPM50 and YPM60 was tested by Fourier Transform Infrared Spectrophotometer (Nicolet-560, Nicolet Co., USA) in the attenuated total reflection mode. The measurements were performed in 400–4000 cm^−1^ wavenumber range with 4 cm^−1^ resolution setting at room temperature. X-ray diffraction (XRD) was used to research the crystallization properties of MA, YP and YPM50 by an automatic diffractometer (Ultima IV, Rigaku, Japan) at 35 kV and 30 mA with Cu Ka radiation. The data were recorded in 5~50° with 0.04°/min scanning rate at 25 °C. The differential scanning calorimetry (DSC) measurements were carried out on DSC Q200 (TA, USA) to find the phase-change temperature and latent heat of Ymer-N120, MA, YP, YPM50 and YPM60 at 10 K/min heating or cooling rate with 5–8 mg. Samples were firstly heated to avoid the effects of the prior processing heat history of samples, then were cooled to obtain the DSC cooling data. Then the next heating step brought the DSC heating performance. Samples were heated and then cooled in 25–80 °C temperature range by 3 K/min heating rate, and the same process was repeated 100 or 500 times in a temperature-controlled chamber. These repeated testing were designed as accelerated thermal-cycles testing. After 100 times or 500 times of testing, these treated samples named YPM50-00, YPM60-00, YPM50-500, YPM60-500. The following heating and cooling cycles of DSC tests were employed to study the phase change temperature and latent heat of thermal treated samples, and to evaluate their thermal reliability. The thermal stability of MA, YP, YPM50 and YPM60 was studied by thermogravimetric analysis (TGA) carried out on a thermal gravimetric analyzer (TGA4000, PE, USA) from 30 to 600 °C at 10 K/min heating rate in nitrogen flow. The microcosmic morphology of fabricated YP and YPM50 was observed by the Field emission scanning electron microscopy (FESEM, Nova NanoSEM450, Thermo Fisher Scientific, USA) with 5.0 kV accelerating voltage.

## Supplementary Information

Supplementary Information 1.

Supplementary Video 1.

## Data Availability

The datasets used and/or analyzed during the current study available from the corresponding author on reasonable request.
