# Supplementary material for: A novel form stable phase change material with comb-like cross-linked polyurethane as supporting skeleton
Source: Sci Rep. 2023 Mar 31;13:5243. doi: 10.1038/s41598-022-21640-3 (PMC10066330; doi:10.1038/s41598-022-21640-3)
Supplement: Supplementary file 1 — Supplementary Information 1. [file 41598_2022_21640_MOESM1_ESM.docx]

**Supporting Information**

**A novel form stable phase change material with** **comb-like cross-linked polyurethane as** **supporting skeleton**

**Yunyun Yang ^a,b^, Shenghua Xiong ^a,b^, Ju Fu ^a,b^, Yuanhua He ^a,b^, Yi Wu ^a,b*^, and Yi Xu ^a,b*^**

^a^: College of Civil Aviation Safety Engineering, Civil Aviation Flight University of China, Guanghan 618307, China
^b^: *Civil Aircraft Fire Science and Safety Engineering Key Laboratory of Sichuan Province, Civil Aviation Flight University of China, Guanghan 618307, China*

***:** *Corresponding authors:* 411619416@qq.com (Yi Wu); xuyi99@cafuc.edu.cn (Yi Xu).

**
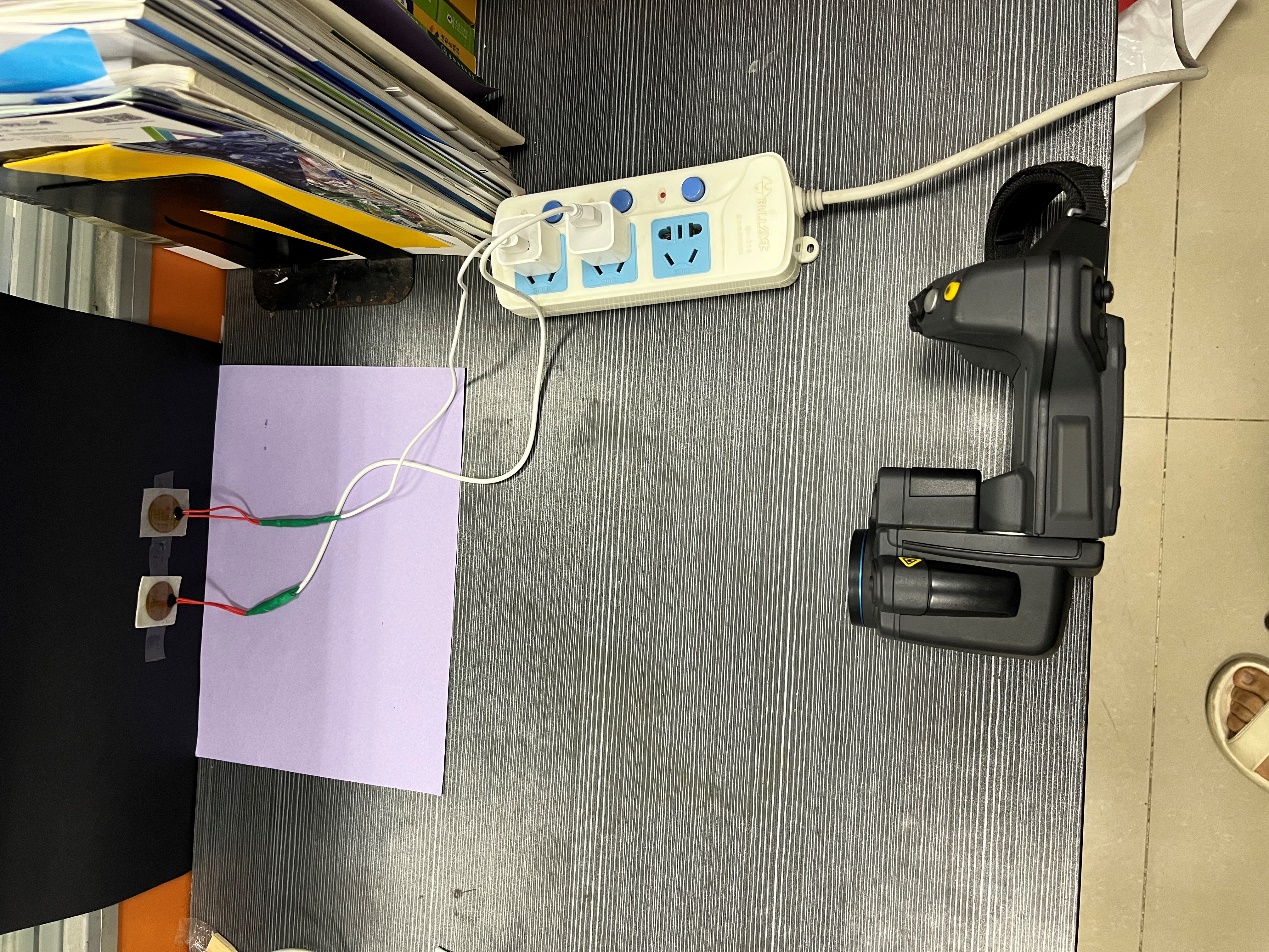
**

**Figure S1.** The device with PI-based heater (50 mm, 2.7Ω) as heat source.


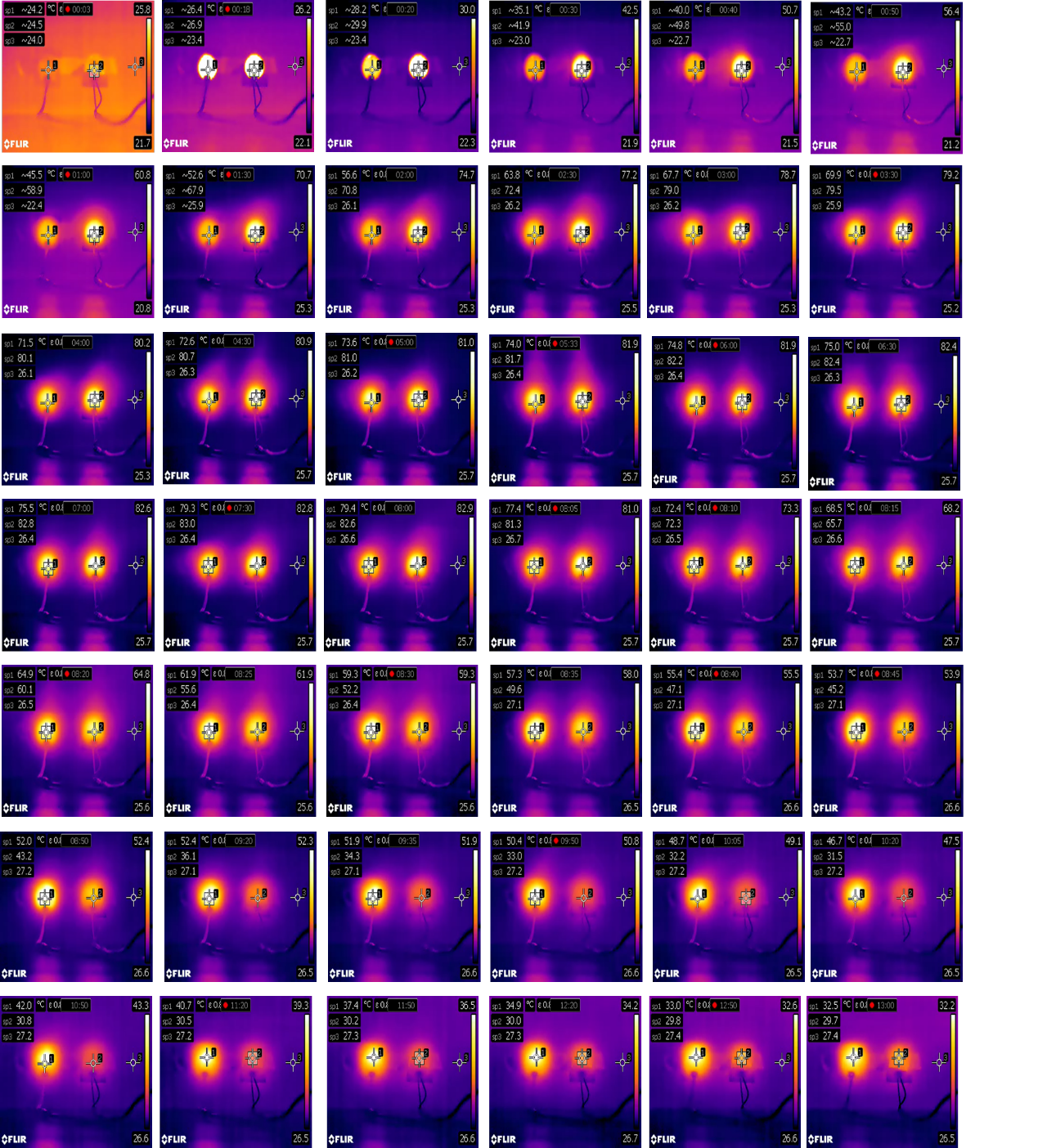


**Figure S2.** The real-time temperature of the heater during use (sp1 is the temperature of PI-based heater with YPM50 film, sp2 is the temperature of PI-based heater with YP film, sp3 is the ambient temperature during testing, the real-time video can also be found in Supplementary Video 1)


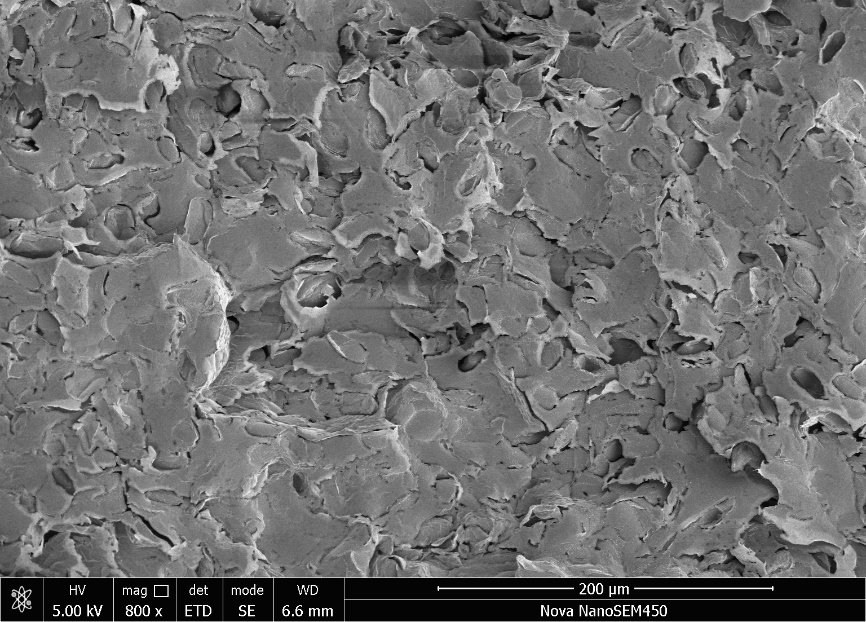


**Figure S3.** the SEM image of YPM50-500.
